# Recognition Properties and Competitive Assays of a Dual Dopamine/Serotonin Selective Molecularly Imprinted Polymer

**DOI:** 10.3390/ijms9122333

**Published:** 2008-11-26

**Authors:** Roongnapa Suedee, Vatcharee Seechamnanturakit, Acharee Suksuwan, Bhutorn Canyuk

**Affiliations:** Molecular Recognition Materials Research Unit, Department of Pharmaceutical Chemistry, Faculty of Pharmaceutical Sciences, Prince of Songkla University, Hatyai, Songkla 90112, Thailand

**Keywords:** Molecularly imprinted polymer, mixed receptor, ergots

## Abstract

A molecularly imprinted polymer (MIP) with dual dopamine/serotonin-like binding sites (DS-MIP) was synthesized for use as a receptor model of study the drug-interaction of biological mixed receptors at a molecular level. The polymer material was produced using methacrylic acid (MAA) and acrylamide (ACM) as functional monomers, *N,N′*-methylene bisacrylamide (MBAA) as cross-linker, methanol/water mixture (4:1, v/v) as porogen and a mixture of dopamine (*D*) and serotonin (*S*) as templates. The prepared DS-MIP exhibited the greatest rebinding of the template(s) in aqueous methanol solution with decreased recognition in acetonitrile, water and methanol solvent. The binding affinity and binding capacity of DS-MIP with *S* were found to be higher than those of DS-MIP with *D*. The selectivity profiles of DS-MIP suggest that the *D* binding site of DS-MIP has sufficient integrity to discriminate between species of non-optimal functional group orientation, whilst the *S* binding site of DS-MIP is less selective toward species having structural features and functional group orientations different from *S*. The ligand binding activities of a series of ergot derivatives (ergocryptine, ergocornine, ergocristine, ergonovine, agroclavine, pergolide and terguride) have been studied with the DS-MIP using a competitive ligand binding assay protocol. The binding affinities of DS-MIP were demonstrated in the micro- or submicro-molar range for a series of ergot derivatives, whereas the binding affinities were considerably greater to natural receptors derived from the rat hypothalamus. The DS-MIP afforded the same pattern of differentiation as the natural receptors, i.e. affinity for the clavines > lysergic acid derivatives > ergopeptines. The results suggest that the discrimination for the ergot derivatives by the dopamine and serotonin sites of DS-MIP is due to the structural features and functional orientation of the phenylethylamine and indolylethylamine entities at the binding sites, and the fidelity of the dopamine and serotonin imprinted cavities.

## 1. Introduction

The ability of synthetic polymers to mimic biological polymers has evolved from the developments in supramolecular self-assembly and living polymerization techniques. These new techniques are utilized in molecular imprinting applications, leading to greater control over three-dimensional organization and recognition specificity [1–5]. Recent significant developments in the use of MIPs for the production of synthetic receptors has led to the application of these materials to a variety of technologies such as separations, chemical analysis, synthetic catalysis, sensors, and drug delivery [6]. MIPs are polymeric materials possessing selective molecular recognition properties due to the presence of recognition sites within the polymer matrix that are complementary to the shape and position of functional groups in the analyte molecule. These extremely stable and robust MIPs lend themselves to the preparation of receptors that are not affected by harsh treatments during the analysis process and which can be used both in organic and aqueous solvents. MIPs are considerably easier to prepare and store than natural receptors from animal tissues. The use of MIPs as adsorbent phases to replace immobilized antibodies in competitive radioligand-binding assays [7] has been extended to the substitution of MIPs for antibodies in fluorescent ligand competitive binding analysis [8], with highly sensitive levels of detection, depending on the fluorescent labels [9, 10].

The important neurotransmitters dopamine (*D*) and serotonin (*S*) are active toward monoamine neurons which are widely distributed in the mammalian central nervous system (CNS). These neurons express specialized plasma membrane transporter proteins that move previously released transmitter molecules from the extracellular space back into the cytoplasm [11]. The neurotransmitters and their receptors play a critical role in both the pathogenesis and treatment of many psychiatric disorders [12]. Several classes of compounds interfere with the neurotransmission at serotonin or dopamine receptor subtypes. The balance between dopamine and serotonin transmission is a vital consideration when developing medications with reduced stimulant side effects due to activation of dopamine neurons in CNS reward circuits [13]. A dual deficit in dopamine and serotonin function occurs during withdrawal from chronic cocaine or alcohol abuse, leading Rothman *et al.* to propose the use of dual dopamine/serotonin releasers for treatment of alcohol and psychostimulant addictions [13]. Furthermore, changes in the balance of dopamine and serotonin (i.e. D_2_ and 5-HT_1A_) receptor interactions profoundly influence the profile of action of some antipsychotic drugs [14].

The enormous complexity of living systems and the separation between cause and effect complicate studies of the relationship between drug administration and pharmacological action. Scientists have sought to simplify the experimental systems by ignoring ancillary factors like drug transport and metabolism and focusing on molecular manipulations and precise physico-chemical methods. Quantitative binding experiments on both whole membrane preparations and on isolated receptors have advanced the understanding of drug-receptor interaction at the molecular level [15], leading to direct experimental access to receptor binding sites and the development of complementary receptor models and the characterization of molecular properties of drug receptors [16]. The objective of the present work has been to prepare molecularly imprinted polymers comprised of dual dopamine and serotonin binding sites as a receptor model for biological mixed neurotransmitter receptors. Dopamine (*D*) and serotonin (*S*), the endogenous dopaminergic and serotoninergic agonists of natural dopamine and serotonin receptors, were used as the printed molecules to generate the molecular selectivity of the synthetic material. Generally, the preparation of MIPs uses single-template imprinting, but recent studies report the synthesis of MIPs containing multiple sites that bind two or even three molecules [17–18]. In a recent effort to introduce multiple-recognition sites into artificial receptors by using a mixture of tetracycline and its degradation products as the multiple templates, Suedee *et al.* noted that the recognition ability of the resulting MIP for tetracyclines was group specific [19]. Imprinting with two or more templates simultaneously rapidly produces a multiple-recognition MIP. In addition, the pre-polymerization complexes of target analytes used as templates are formed under the same conditions, allowing consistency of the imprint.

Ergots, a type of ergoline derivatives [20–23] consisting of adrenergic, dopaminergic and serotonergic pharmacophores, were the test compounds of this study. Their pharmacological activities are mediated through binding to adrenergic, dopaminergic, and serotonergic receptor sites. Ergot compounds may act as partial agonists or antagonists at the receptor sites of biogenic amine neurotransmitters which mediate a broad spectrum of pharmacological activities, including central, peripheral, and neurohumoral effects. This activity has many important clinical implications, such as treatment of Parkinson’s disease and hyperprolactinemia [24, 25]. Natural dopamine and serotonin receptors from different sources, including the brain (striatum or hypothalamus) of sacrificed rats, other rodent species [26], monkey [27], or from preparations of cloned human receptors [28] have been used for determining ligand-binding characteristics of ergot derivatives.

**Figure 1.**
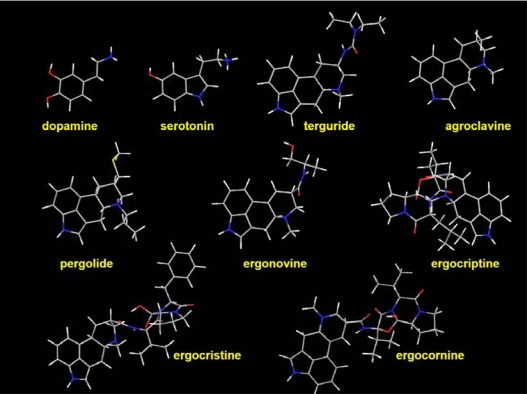
The MM2 structures of dopamine, serotonin, and ergot derivatives used in this study (Chem3D Ultra 10.0, CambridgeSoft, MA, USA); *grey = carbon, white = hydrogen, red = oxygen, blue = nitrogen, yellow = sulfur*.

In this study, the dual dopamine/serotonin-selective molecularly imprinted polymer (DS-MIP) was synthesized by the interaction between the *D* and *S* template and functional monomers, MAA and ACM, together with cross-linking polymerization of MBAA. Two single-recognition MIPs; dopamine imprinted polymer (D-MIP) and serotonin imprinted polymer (S-MIP) were prepared to serve as reference polymers. The MIPs prepared as a bulk monolith and transformed into granules were characterized physically, and their ability to rebind *D* and *S* was assessed in various solvents: acetonitrile, water, methanol and methanol/water mixture. Based on the binding results, the solvent that conferred the best selectivity for MIPs was identified and then used to study the binding characteristics of ligand in competitive binding assay using DS-MIP. In the present work, we have studied the recognition properties of the prepared DS-MIP and its application as a selective adsorbent phase for the determining ligand-binding characteristics of ergot-related compounds (Figure 1) toward the dopamine/serotonin binding sites. By comparison with the results from a competitive immunoassay, obtained using dopamine/serotonin receptors derived from rat hypothalamus the novel DS-MIP have proven to be selective sorbent for identifying ligand of dopaminergic, and serotonergic receptors of biological receptor.

## 2. Results and Discussion

### 2.1. Synthesis and characterisation of DS-MIP

**Figure 2.**
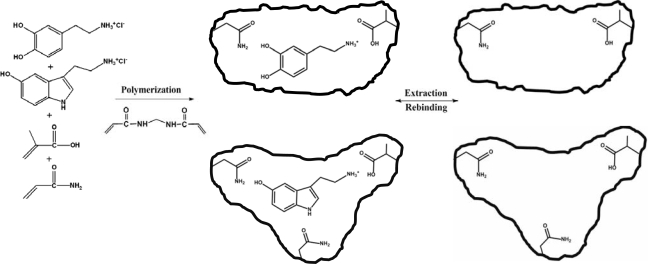
Possible structures of the molecularly imprinted polymer using *D* and *S* as templates.

The dual dopamine/serotonin-selective molecularly imprinted polymer was successfully prepared by thermal polymerization using a mixture of *D* and *S* as mixed templates. A mixture of methanol and water (4:1, v/v) was chosen as the porogen solvent for the *D* and *S* templates, as the hydrochloride salts were soluble only in polar solvents. The strength and positioning of the monomer-template interactions are of importance in developing materials with good molecular recognition properties. For the current work, the functional monomers and cross-linker had been constructed into the MIP structure with a view toward obtaining an efficient recognition system in a polar medium. MAA and ACM were generally used as functional monomers for the imprinting process [29, 30]. Both *D* and *S* have a phenolic hydroxyl and the nitrogen atom(s) of the amine group(s) acts as a hydrogen donor-acceptor moiety for binding functional monomers with a corresponding hydrogen bonding pattern. Hyperchem molecular modeling studies suggested H-bond interactions between the −COOH of MAA and the template molecules’ −NH_2_, the −NH of ACM and the oxygen atoms of the phenolic hydroxyl group on the template molecules, and between the C=O of ACM and the nitrogen atoms of the five-membered ring on the *S* molecule. However, the huge excess of water and methanol that would be form H-bonds in competition with template and monomer, would reduce binding energy between template and monomer. Hence, MAA and ACM functional monomers were tested for their ability to generate specific binding sites in d_6_-DMSO by a ^1^H-NMR titration. Based on changes in the chemical shift of the protons in d_6_-DMSO, the 1:1 complexation of *D* with the monomers probably involves the respective amide or carboxylic acid of ACM and MAA hydrogen bond interactions with the hydroxyl group of *D*. The apparent dissociation constant (app. *K*_diss_) of the complex formation between *D* and the single monomer was found to be 1.98 ± 0.1 mM for ACM and 2.12 ± 0.2 mM for MAA. For the *S* template, the spectral shifts due to hydrogen bonding were identified in the NMR spectrum. For ACM, the app*. K_diss_* values of 0.19 ± 0.1 mM and 0.43 ± 0.1 mM for the template’s hydroxyl and indolyl amino groups respectively, were identified, while app*. K_diss_* values of 0.68 ± 0.1 mM and 0.92 ± 0.1 mM were estimated for the 1:1 complex formation between MAA and the hydroxyl and indolyl amino groups of the template, respectively. Such values would suggest that both MAA and ACM would form a strong complex with the template(s) molecule in polar solvents. NMR confirmed that the carboxylic acid or amide group of MAA and ACM is likely to form hydrogen bond with the hydroxyl or indolyl amino groups of the template molecule(s) though the H-bonding formation between the carboxylic acid group of MAA and the primary amino group of the templates that was postulated by Hyperchem was not identified by NMR. This is likely to be due to the NH_3_^+^ of the salt of primary amine of the templates which might form electrostatic interactions or weak hydrogen bond with the carboxylic acid of MAA in aprotic solvent (d_6_-DMSO). The NMR results suggested the higher level of the pre-polymerisation monomer/template complex for *S* rather than *D*. MBAA was employed as the cross-linking monomer for imprinting polymer with *D* and *S*, since it provides a high flexibility and conformational adaptability to the polymer, and thereby partially mimic the properties of the protein-based natural recognition systems. In previous work, the use of MBAA as cross-linking monomer provided a high degree of selectivity for polymer imprinting with *D* in the presence of the functional monomers, as was the case in this study when using aqueous methanol as solvent [31]. The MBAA cross-linker also forms hydrogen bonds with the carboxylic acid and amino groups of the templates [30]. Figure 2 illustrates the dual dopamine/serotonin molecular recognition site generation in this work.

**Figure 3.**
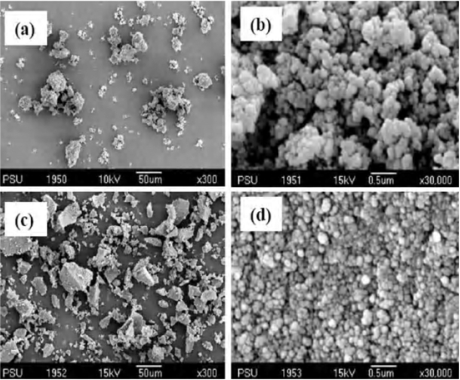
SEM micrographs of DS-MIP × 300 (a), × 30,000 (b) and non-imprinted polymer × 300 (c), × 30,000 (d).

A study of certain features of the polymer can yield information important to the physical characteristics of rebinding. The polymer produced with the use of methanol and water as the porogenic environment was examined by size distribution by BET enabled the nature of the size and distribution of the pores within the polymer matrix to be probed. The imprinted and non-imprinted polymer (NIP) materials prepared had particle sizes ranging from 20–30 μm (Table 1), as determined by a laser diffraction apparatus (Malvern Mastersizer, Worcester, UK), with the size of the NIP particles being slightly larger than the MIPs. The morphologies of DS-MIP and NIP are irregularly shaped particles (see Figures 3a,c). Their surfaces are composed of microspheres or aggregates of microspheres aligned on the surface of the irregular shaped particles (Figure 3b,d). The surface of DS-MIP was composed of aggregates of packed primary particles in the size range 100–200 nm (see Figure 3b) and the surface of NIP was comprised of numerous microspheres smaller than those of DS-MIP (Figure 3d), suggesting a larger surface area of NIP than for MIP. Analysis of pore sizes shows that the polymer powders contain micropores (< 5 nm) and mesopores with maximum pore diameters of 100 nm. The micropores and mesopores are ascribed to the interstitial voids of the packed primary particles within the aggregates. The macropores come from the interstitial space formed among the microparticles and aggregates. The polymers swelled in pure methanol and pure water, and in a mixture (4:1, v/v) of methanol: water, they swell approximately 80–100%, as measured by inspection using a graduated cylinder.

The pore diameters and pore volumes of the imprinted and corresponding non-imprinted polymers were inspected by nitrogen adsorption/desorption techniques using a Coulter SA3100 series surface area and pore analyzer (Beckman Coulter, CA, USA). The pore diameters of the imprinted polymers were smaller than those of the non-imprinted polymer while the pore volumes of all preparations were similar, varying over the narrow range of 0.21–0.31 mL/g (Table 1). The specific surface area and pore size of DS-MIP and the single-recognition MIPs were lower than those of the control. This result is consistent with the previous reports of MBAA-based MIPs with thermoresponsive properties [31] in which the mean pore size and specific surface area were lower than those of the control polymer prepared in the same manner as MIP but omitting template. This result in the MBAA-crosslinked gel polymers was attributed to compression of the template cavities within the gelling network that occurred as the temperature decreased from the polymerizing temperature (60 °C) to the extraction temperature (room temperature). The D-MIP was prepared using the same experimental conditions than S-MIP but with the only difference of template employed. The DS-MIP has double concentration of templates used in the polymerization recipe versus D-MIP as well as S-MIP. The pre-polymerization complexes of the *D* and *S* templates are formed under the same conditions except the concentration of the templates used in the polymerization recipe was higher than the reference polymers. It is apparent that the specific surface area and pore volume of S-MIP were higher than those of D-MIP and DS-MIP. The higher pore volume and surface area of S-MIP suggested higher capacity and better formed pores, which is more likely to allow a higher rate of rebinding than those of the D-MIP. The average pore size diameter of D-MIP was higher than that of DS-MIP, but that the specific surface area and pore volume of these polymers were not significantly different. On the other hand, the average pore size diameter, and the micropore surface and pore volume of DS-MIP and S-MIP did not differ. Studies have demonstrated the issues involved with single template-template interactions, which in case of the template molecules can result in aggregation inside the pores of the MIP and this might lead to polymer performance and morphology being modified. However, it is shown that there is still a relatively large population of pores in the micro- or lower mesoporous range.

### 2.2. Template recognition by DS-MIP

**Figure 4.**
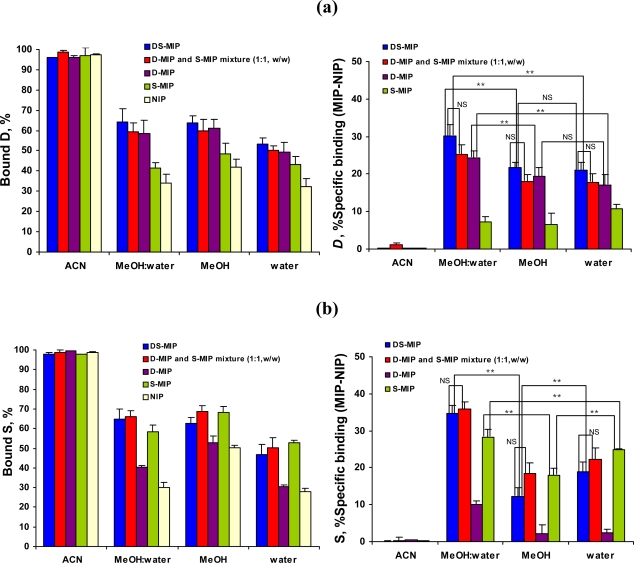
Effect of solvent on the binding of dopamine (*D*) and serotonin (*S*) to the imprinted and non-imprinted polymers. Batch binding studies using 50 mg of polymer particulate and 25 μg of ligand in a final volume of 5 mL (*D* = 26.40 μM, *S* = 23.50 μM) were performed at room temperature as described in the Experimental Section. The values shown are mean ± SD of 3 or 5 replicates. Panel (a), binding of *D*; panel (b), binding of *S*. (NS) indicates no significant difference, (**) p < 0.05.

We evaluated template recognition by DS-MIP by batch binding analyses in parallel experiments with the reference polymers (D-MIP, S-MIP and the D-MIP/S-MIP (1:1, w/w) mixture). Binding studies with the non-imprinted polymer (NIP) determined non-specific binding of the polymers. Figures 4(a,b) show the binding of *D* and *S* ligands to DS-MIP, and to the reference and the non-imprinted polymers, when incubated in acetonitrile, methanol, water, and a mixture of methanol/water (4:1, v/v), the solvent used for the synthesis. Template recognition by DS-MIP and all the reference polymers is highest in the methanol:water mixture, confirming that the synthesis medium for the MIPs was an appropriate solvent for selective binding of *D* and *S* ligands. The binding results with the DS-MIP were comparable to those obtained for the single-recognition MIPs, D-MIP and S-MIP.

The recognition ability of DS-MIP was similar to the D-MIP/S-MIP mixture in all circumstances, suggesting few template-template interactions of the two templates occurred during pre-polymerisation. Given the higher degree of complexation of the template and due to the greater accessibility of the functional monomers, the S-MIP was expected to show a higher degree for selectivity of template than the D-MIP.

The binding of the *D* and *S* templates was highest in acetonitrile, and lowest in water (Figure 4). Likewise, the non-specific adsorption of the *D* and *S* templates was high in acetonitrile. With acetonitrile as solvent, both *D* and *S* templates bind extensively (about 95%, non-specifically) to all the polymers. With methanol, water, and the methanol/water mixture, such non-specific binding to NIP was reduced by 35–40% in all solvents. The % specific binding of MIPs for *D* ligand was in the order methanol/water mixture > methanol ~ water >> acetonitrile, whereas for *S* ligand the binding was in the order methanol/water mixture > water > methanol >> acetonitrile. Methanol and water appeared to affect to the rebinding of *D* and *S* ligands to the MIPs differently. Methanol induced an increased non-specific adsorption of the MBAA-polymer with respect to other solvents, due to increase in an H-bonding. When water was used, the selectivity was not improved though non-specific binding decreased. Both the binding assay and NMR results indicated that the interactions probably involve the carboxylic acid and amide of MAA and ACM acting as anchor monomers, forming an electrostatic interaction to the template ethylamine moiety or forming hydrogen bonds with the template hydroxyl or/and indolyl amine moieties. Results indicated that the MIPs possessed a high degree of cross-reactivity for close analogs of the template. The MIPs perform well in terms of rebinding when used in the solvent they were manufactured since it is likely that the links generated between the domains of the structurally flexible MBAA-MIPs under such conditions are optimal for template-function site interaction. The results suggested that the selectivity arises from a combination of electrostatic interaction and H-bonding of the templates to both the functional monomer(s) and crosslinker. Selective adsorption was confirmed by comparison to the control polymer. Comparison of the recognition properties of the DS-MIP to those of each single-recognition MIP, dopamine imprinted polymer (D-MIP) and serotonin imprinted polymer (S-MIP), and the reference (D-MIP/S-MIP mixture) indicated the specific interactions due to the presence of binding sites, also verified the template-template interaction of the two templates employed may occur during pre-polymerisation process.

### 2.3. Binding characteristics of DS-MIP

The binding affinity of DS-MIP was evaluated in binding experiments along with the reference polymers (D-MIP and S-MIP). These experiments confirm the selectivity of DS-MIP and the presence of specific binding sites in its structure, making it suitable for performing rebinding experiments. To perform this study, solutions of *D* and *S,* separately, at concentration ranging between 0.25 to 105 μM were incubated with 100 mg samples of polymers and the concentration of bound (*B*) and free (*F*) substrate were calculated as described in Section 3. The binding constants were measured with the binding capacities for the MIPs by extrapolation of data measurement of *D* and *S* concentration ranging from 0.04 to 53 μM. Scatchard plots of the ratio of bound *D* (or *S*)/freely soluble versus bound *D* (or *S*) revealed the presence of multiple binding sites. Table 2 shows the binding constants for the imprinted and non-imprinted polymers. The binding affinity of *D* and *S* to the non-imprinted polymer is very low in all cases. The results suggest that the MIPs have cross-reactivity toward compounds that have structural similarities to the template(s). The binding results obtained in the present study show that the selectivity of *D* and *S* molecules for DS-MIP is mainly due to polymer imprinting with the mixture template.

The results obtained from the Scatchard analysis demonstrated that the recognition sites of the MIPs are heterogeneous. An appropriate approach is needed to use to describe binding as case of the MIPs. It is important to make the comparison of the binding properties (i.e. capacity, affinity constants, heterogeneity) of DS-MIP and the single imprinted polymers, since this enables to understand how molecular recognition event takes place. Umpleby *et al*. [32, 33] have recently demonstrated that the Langmuir–Freundlich isotherm (LF) is able to address the heterogeneity of MIPs in both covalent and non-covalent approaches and in sub-saturated and saturated zones. The Langmuir–Freundlich (LF) isotherm describes a relationship between the concentration of bound (*B*) and free (*F*) guests in heterogeneous systems according to Eq. (1):

(1) $$B=\frac{N_{t}aF^{m}}{1+aF^{m}}$$

where *N_t_* is the total number of binding sites (capacity), *a* is related to the median binding affinity constant *K*_o_ (*K*_o_ = *a^1^*^/^*^m^*) and *m* is the heterogeneity index. The LF fitting coefficients *N_t_*, *a* and *m* yield a direct measure of the total number of binding sites, mean association constant (via *K*_o_= *a^1^*^/^*^m^*), and heterogeneity, *m* is the heterogeneity index which will take values between 0 and 1. When *m* is closer to 1, the material presents a more homogeneous binding site distribution. This model allows the direct measurement of the fitting parameters, which can be used either for the comparison of different MIPs [32] or to study the adsorption of different compounds to the same MIP [34]. The experimental adsorption isotherms obtained for DS-MIP, D-MIP and S-MIP had been fitted to the LF isotherm and the binding coefficients obtained are shown in Table 3. It is noted that the excellent *R*^2^ coefficients were obtained when the LF isotherm was used confirming the suitability of this isotherm to model the interactions taking place in MIPs.

Either the MIP prepared with the template mixture or MIP prepared by using the single template showed the greater number of binding sites of high affinity and specificity for the *S* template in comparison to the *D* template. These results can be only attributed to the presence of better-defined binding sites that would be able to strongly interact with the *S* template in the MIPs. This result could be explained by taking into account that the monomer-template complex formation and the stability of the pre-polymerization complexes of the structurally rigid *S* template which provided the multiple sites for interaction with the functional monomers were higher than those for *D* template. The capacities obtained for *D* and *S* templates in DS-MIP were slightly higher than those shown by D-MIP and S-MIP. This result suggests that the presence of the same proportion of *D*-, and *S*-structure selective sites in the DS-MIP though *D* and *S* are able to interact to these binding sites and enabled the slight increase in the capacities of DS-MIP for *D* and *S*. The high *N_t_* was observed for *S* and *D* in the single imprinted polymer D-MIP and S-MIP, respectively, but that lower *K*_o_ value was obtained for the compound studied, suggesting that *S* and *D* interact with the additional binding sites present in D-MIP and S-MIP. It has been shown that the capacity for *S* in D-MIP is higher than the capacity for *D* in S-MIP whilst the *K*_o_ value is reverse case. These results might suggest that the strength of interaction of the close analog of template with some binding sites in the reference polymers may be higher than the given analyte for the binding sites in DS-MIP. Thus, apparently, the amount of this kind of binding sites would very low in DS-MIP and hence the *K* values associated to them would have a less impact in the calculation of *K*_o_ value. In comparison of *m* values of the MIPs, the D-MIP shows a more homogeneous binding site distribution versus S-MIP. The same trend was observed for degree of binding site homogeneity present in DS-MIP and the single-recognition MIPs, when the templates were examined independently.

### 2.4. The specificity of DS-MIP

**Figure 5.**
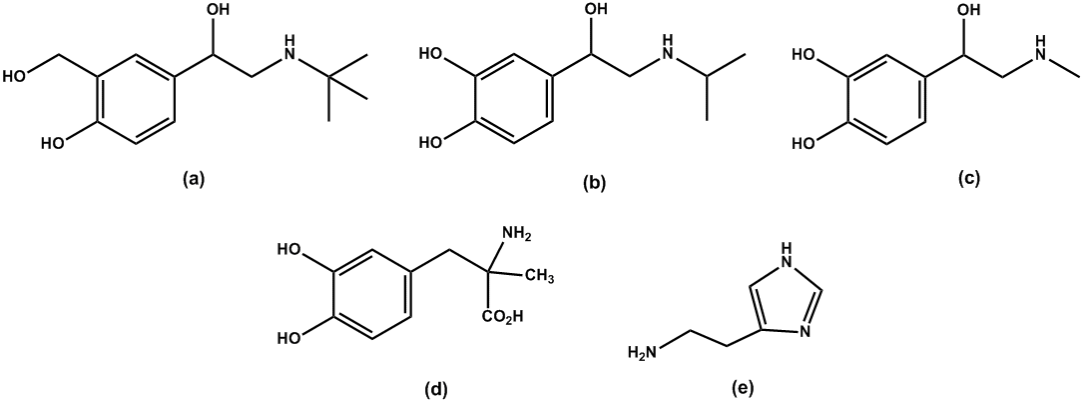
Molecular structures of drugs used to study the selectivity of DS-MIP: (a) salbutamol, (b) isoproterenol, (c) epinephrine, (d) methyldopa and (e) histamine.

**Figure 6.**
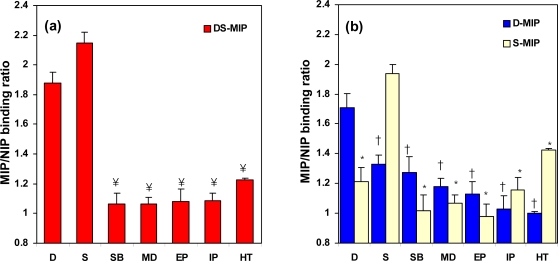
Percentage binding of various adrenergic ligands and serotonergic ligands for *D* and *S* binding sites on: (a) DS-MIP and (b) the single-recognition MIPs (D-MIP and S-MIP) at room temperature (28 ± 1°C). Binding studies were conducted in 4:1 (v/v) methanol/water using 50 mg of polymer and 25 μg of each ligand in a final volume of 5 mL (21.0-45.0 μM). At equilibrium, analytes were quantitated in the filtrate by UV or fluorometric assays as described in the Experimental Section. The values shown are mean ± SD of 3 or 5 replicate specimens. (¥) p < 0.05 versus *D*/*S*; (†) p < 0.05 versus *D*; (*) p < 0.05 versus S. Dopamine (*D*), serotonin (*S*), salbutamol (*SB*), isoproterenol (*IP*), epinephrine (*EP*) methyldopa (*MD*), histamine (*HT*).

The specific selectivity achieved with DS-MIP was further examined to ascertain its recognition ability for dopamine, salbutamol, epinephrine, isoproterenol, methyldopa, serotonin, and histamine (Figure 5). The DS-MIP showed higher specific binding for the *D* and *S* template(s) than the other structurally similar compounds (Figure 6a). S-MIP appears to recognize the *S* template much better than the structurally similar compounds (Figure 6b). On the other hand, D-MIP not only presents good recognition of the *D* template, but it also has reasonable cross-reactivity with the compounds that are structurally similar to the template (see Figure 6b). The cross-reactivities shown by D-MIP for these ligands indicate that a basic structural motif is sufficient for recognition. Since the common structural feature of these ligands is the phenylethylamine unit, substituents on this structure appear to lead to the observed variations in recognition.

Selective recognition ability of DS-MIP, D-MIP and S-MIP for the print molecule(s) with a phenylethylamine structure was higher than for molecules with a β-hydroxyl group on the phenylethylamine unit. The steric constraints imposed by the hydroxyl group in the three-dimensional arrangement of the compounds may account for this difference, such as the less favorable interaction that occurs when the β-hydroxyl group is present. Histamine, with an imidazole ring, favorably binds to S-MIP, but has less affinity for D-MIP, indicating that a catechol structure promotes ligand binding to D-MIP, whereas this structural feature is not crucial for binding to S-MIP. The cross-selectivity exhibited by S-MIP for dopamine and histamine ligands suggests that the *S* imprinted cavity could easily accommodate the smaller dopamine, or histamine molecules, and the orientations of the functional groups within the cavity complement those of either dopamine or histamine. A significant loss of selectivity of D-MIP was observed for analogues exhibiting orientational differences to the *D* template, indicating the overall significance of structural affinity. The higher cross-selectivity of D-MIP for *S* than that of S-MIP for *D* suggests that the specificity of D-MIP/DS-MIP may be highly selective to putative structural variations in the size of the binding ligand(s). The results presented in this study demonstrate that the *D* binding site of DS-MIP/D-MIP has sufficient integrity to discriminate between species of non-optimal functional group orientation, whilst the *S* binding site of DS-MIP/S-MIP is less selective toward species having structural features and functional group orientations that differ from *S*.

### 2.5. Specificity of the ergot derivatives towards DS-MIP

**Figure 7.**
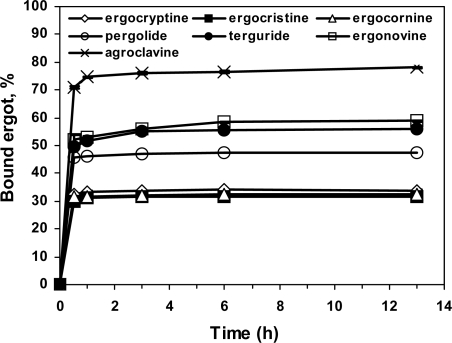
Time course for the binding of ergot compounds to DS-MIP at room temperature (28 ± 1 °C). Binding studies were conducted in 4:1 (v/v) methanol/water using 12.5 mg of polymer and 12.5 μg of each ergot in a final volume of 5 mL (4.1–10.5 μM). At equilibrium, analytes were quantitated in the filtrate by chromatographic assays as described in the Experimental Section. The values shown are mean ± SD of three replicate specimens.

The specificity of binding of a group of ergoline derivatives (ergocryptine, ergocornine, ergocristine, ergonovine, agroclavine, pergolide and terguride) to DS-MIP was assessed in 4:1 (v/v) methanol/water at room temperature (28 ± 1 °C). The binding of all the ergots to DS-MIP reached a constant value within 3 h, with ~50% of the DS-MIP-analyte complex being formed by 1 h (Figure 7). More than 95% of the equilibrium concentration for the ergot derivatives was achieved at a DS-MIP concentration of 2.5 mg/mL, and an incubation time of 6 h. Agroclavine exhibited the greatest binding values (70%) with DS-MIP, while ergocryptine, ergocristine, and ergocornine showed the lowest binding interactions, about 2 times lower than agroclavine. Intermediate levels of binding were observed with pergolide, terguride and ergonovine, ~ 1.25 fold lower than that of agroclavine. Based on the specific binding of the ergots to DS-MIP, the tested ergots are readily distinguished into three groups, based on their chemical structures. All of the ergot derivatives tested had typical four fused rings, indolyl amine, piperidinyl amine, and *N*-methyl and heteroatom-containing alkylpiperidinyl substituents (except pergolide with the *N*-propyl substituent). The differential binding of the ergot derivatives is probably a consequence of the interaction of the piperidinyl and indolyl amines with acidic and amide functions within the polymer. The orientation of functional entities within the *D* and *S* imprinted cavities was not optimal to complement the ergot derivatives, but they existed in sufficient numbers to produce the observed binding effect.

### 2.6. Affinity of the ergot derivatives towards DS-MIP

**Figure 8.**
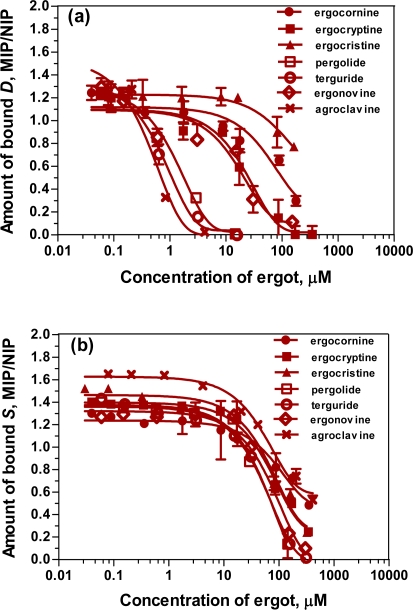
Plots of the ratio of the normalized signal (*B*/*B***î**) for MIP to that obtained for NIP against the logarithm of the ergot concentrations for the competitive ligand binding assay using DS-MIP as adsorbent phase and (a) *D* and (b) *S* as a fluorescent probe. The values shown are mean ± SD of three replicate specimens.

In this study, DS-MIP was employed as the sorbent phase for the determination of binding characteristics of the ergot family of alkaloids by competitive ligand binding assay experiment. The ability of the various ergot alkaloids (ergocryptine, ergocornine, ergocristine, ergonovine, agroclavine, pergolide and terguride) to displace bound *D* and *S* from the binding sites was measured. Figures 8(a,b) show competitive binding assays with the ergots in the presence of *D* and *S* as fluorescent probes. The typical sigmoid calibration curves were obtained for both the *D* ligand and the *S* ligand. As the amounts of the ergot derivatives are increased, increased displacement of the *D* and *S* probes from the DS-MIP is observed, thereby demonstrating the reversibility of ligand-receptor interaction on DS-MIP. Clearly, the ergot derivatives are able to act as competitors at the DS-MIP binding sites. The apparent inhibition constants (*K_i_*) obtained from these experiments are listed in Table 4.

The binding affinities of DS-MIP for all the tested ergots were modest for both *D* and *S* receptor displacement characteristics (Table 4). There were, however, differences for the ergot derivatives for competitive binding with *D* and *S* sites on DS-MIP. The *K_i_* values of the ergopeptines (i.e. ergocornine, ergocristine and ergocryptine) with respect to *D* were 2 - 5 fold lower than the lysergic acid derivatives of the acid amide type (i.e. ergonovine), which in turn had affinity about 150 - 500 fold lower than the clavine derivatives (i.e. agroclavine, pergolide and terguride) for both the *D* and *S* binding sites. Markedly tighter binding of agroclavine, pergolide and terguride to the *D* binding sites was noted in contrast to the *S* binding sites. All the ergopeptines displayed poor affinity at both *D* binding sites and *S* binding sites, as compared to the other compounds. In general, the ergopeptine derivatives and the lysergic acid derivatives of the acid amide type bound to *S* binding sites with higher affinities than those bound to *D* binding sites. The ergot derivatives studied only differ in the heteroatom-containing alkyl group at the C-3 piperidine of the four fused rings, and this structural difference relates to the differential affinities achieved for the ergot derivatives. The energy minimised 3-D structures of *D*, *S*, and the ergot derivatives (see Figure 2) show the structural feature(s) and orientation of the phenylethylamine moiety and/or indolylethylamine moiety that interacts with functionalities of the imprinted polymer. Neither ergocornine nor ergocristine nor ergocryptine adopt a conformation like the phenylethylamine or indolylethylamine moiety of either *D* or *S,* which agrees with the observation that these three compounds have the lowest capability to displace *D* or *S* from their binding site. Of all the ergot derivatives studied, the clavine derivatives (pergolide, terguride, agroclavine) produced the greatest competitive binding for both the *D* and *S* binding sites. Pergolide and terguride, or agroclavine and ergonovine (lysergic acid derivatives) show 3-D structures similar to the benzyl and indolyl moieties of the four fused rings, but have dissimilar orientation of the piperidinyl nitrogen moiety, and this feature enables the *D* and *S* imprinted sites to distinguish these amino moieties.

Binding affinities with the DS-MIP mimic receptor are in the micro- or submicro-molar range, whereas the binding affinities are considerably greater to natural receptors derived from the rat hypothalamus (see Table 4). The natural receptor differentiates the above compounds into three groups. The *K_i_* values with the DS-MIP are much higher than for the natural receptor, but nonetheless the artificial MIP receptor data reveals the same pattern of differentiation i.e. affinity for the clavines > lysergic acid derivatives > ergopeptines.

The competitive assay using DS-MIP demonstrates that all the ergots studied act at the dopamine/serotonin binding receptors and that the binding characteristics of the compounds show a similar pattern of differentiation with both the DS-MIP mimic and the natural D/S receptors (Table 4). The findings confirm the ability of pergolide and terguride to specifically bind to dopamine receptors, in good agreement with the result obtained with the rat hypothalamus receptor (see Table 4). The results obtained from the binding study using rat hypothalamus receptors were in agreement with those of previous studies [28, 35, 36]. The competitive binding assay, using the DS-MIP material as an adsorbent phase and dopamine/serotonin as fluorescent probes, holds great promise as a tool to identify ligands of natural dopamine and serotonin binding receptors.

## 3. Experimental Section

### 3.1. Chemicals

Dopamine hydrochloride, serotonin hydrochloride, isoproterenol, salbutamol sulfate, histamine, methyldopa, epinephrine, agroclavine, ergocryptine, ergocornine, ergocristine, ergonovine maleate, pergolide mesylate and terguride were obtained from the Sigma-Aldrich Chemical Company (Milwaukee, WI, USA). *N,N′*-Methylenebisacrylamide (MBAA), methacrylic acid (MAA) and acrylamide (ACM) and sodium heptanesulfonate were purchased from Sigma-Aldrich Chemical Company (Milwaukee, WI, USA). 2,2′-Azobis(isobutyronitrile) (AIBN) was purchased from Janssen Chimica (Geel, Belgium). MAA was purified by distillation under reduced pressure before use. Working standard solutions were prepared fresh daily. All solvents were of either analytical or HPLC grade.

### 3.2. Apparatus

The molecular modeling interactions of the templates and the functional monomers were studied with a Hyperchem 7.5 software package (Hyperchem Inc, Gainesville, FL, USA) using the Amber MM method at the HF/3-21G* level using a small basis set (HF/3-21G* level). Proton NMR spectra were obtained on Varian 500 MHz FT-NMR spectrometer (Varian, CA, USA). UV absorbance measurements and spectra were recorded using a Hewlett-Packard diode array spectrophotometer Series 8452A (Hewlett-Packard, CS, USA). Fluorescence measurements were performed with a LS50B Perkin Elmer luminescence spectrometer equipped with a 150 W xenon lamp (Perkin Elmer, Shelton, CT, USA). An Agilent 1100 HPLC system (Hewlett-Packard, CS, USA) consisting of a G1322A quaternary pump, G1322A in-line solvent degasser, G1313A auto injector (20 μL injection loop), equipped with a Hewlett-Packard 1049A programmable electrochemical detector by a 35900E Hewlett-Packard interface was used. HPLC data were collected and analysed on a personal computer using HP ChemStation (Hewlett-Packard, CS, USA).

### 3.3. Investigation of template/functional monomer complexation by ^1^H-NMR spectroscopy

The change in chemical shifts of proton resonances as a function of *D* (or *S*) concentrations was studied in DMSO-d_6_. Ten milligrams of *D* (or *S*) was titrated with aliquots of ACM (or MAA) by physical mixing using a pestle and mortar. A ^1^H-NMR spectrum of the sample mixture (2 mg) in DMSO-d_6_ (500 μL) was examined at 25 °C. The peak at 2.6 ppm assigned to the solvent was considered as an internal standard in the measurement of the chemical shifts of the peaks of the compounds. The *D* (or *S*) for the mixtures was then compared by the superimposition of the spectra. The peak difference between these *D* and *S* sets were calculated and plotted as a function of functional monomer amount in order to obtain the saturation isotherms and the apparent complex dissociation constants (app. *K_diss_*) were calculated from these plots.

### 3.4. Polymer synthesis

The DS-MIP was synthesised by *in situ* polymerization of the template, MAA, ACM and MBAA at 2:2:2:10 molar ratio; a 1:1 molar ratio of *D* and *S* mixture was used as the template. The molar ratio of template: MAA:ACM:MBAA for single-recognition MIPs (D-MIP and S-MIP) was kept as 1:2:2:10. The polymers were synthesised as reported earlier [31] using a method outlined by Vlatakis *et al.* [7] using thermal polymerization with AIBN as an initiator. The monomeric components were dissolved in a methanol/water mixture (25 mL, 4:1, v/v) along with the initiator. Subsequently, the mixture was purged with a stream of nitrogen gas for 5 min to remove the radical scavenger oxygen. Copolymerization was carried out at 60 °C for 18 h in a hot-air oven. The resulting polymers were crushed, ground, and sieved through a 100 mesh-sieve having an open width of 150 μm. The polymer particles were washed with portions of 10% v/v acetic acid in methanol (3 × 500 mL), and finally with portions of methanol (3 × 500 mL). Complete extraction of the template molecule from the polymer was confirmed by the absence of the template(s) in the methanol rinses of polymer, as verified by a fluorescence spectroscopic method. Finally, the polymer particles were dried under vacuum and stored in air tight containers at ambient temperature until use. The polymers imprinted with *D* from the same batches as that in the earlier work [31] were employed. A non-imprinted polymer (NIP), which was incorporated as the control, was prepared in the same way as DS-MIP, except that the template molecules were omitted.

### 3.5. Determination of the selective recognition ability of DS-MIP

The recognition ability to *D* and *S* by DS-MIP and the reference polymers (i.e. D-MIP, S-MIP and 1:1 (w/w) D-MIP/S-MIP mixture) were evaluated in acetonitrile, water, methanol, and a methanol/water mixture (4:1, v/v), using batch binding assays. Control experiments were carried out with the NIP. In a typical binding experiment, the polymer particulate (50 mg) was added to the solvent (5 mL) containing dopamine (5 μg/mL, 26.40 μM) (or serotonin, 23.50 μM), or the pure solvent (blank, 5 mL), and the suspension was stirred for 24 h at room temperature (28 ± 1 °C). The polymer particles were then filtered, and the filtrate analysed for free dopamine or serotonin content by fluorescence spectrophotometry, using 279 nm and 320 nm as the excitation and emission wavelengths, respectively, for dopamine analysis, and 300 nm and 335 nm as excitation and emission wavelengths, respectively, for serotonin analysis [31]. The quantity of the drug in the solution was determined by reference to a calibration curve. The amount of bound drug was obtained by subtracting the amount of free drug from the total amount of the drug added. The binding of *D* and *S* templates were calculated and are reported as percentage binding values. All experiments were performed in triplicate.

### 3.6. Determination of the binding characteristics of DS-MIP

The binding affinities of DS-MIP, D-MIP, S-MIP and NIP were evaluated on 50 mg samples of polymer with template solutions ranging in concentration from 0.05 to 200 μg/mL (0.25–105 μM), and methanol/water (4:1, v/v) mixture as the medium at room temperature (28 ± 1 °C). The amount bounds of *D* and *S* on the MIPs were quantified from the non-bound fractions. The values of amount bound were plotted against the concentrations of *D* and *S*. The binding constants were determined from the equation:

(2) $$\text{Bound}/\text{Free}=(B_{\text{max}}-B)/K_{d}$$

where *K_d_* is the equilibrium dissociation constant, and *B_max_* is the maximum number of binding sites [37]. The mean drug binding constants were calculated from triplicate independently derived results.

The total binding capacities and degree of multiplicity of binding sites of the MIPs were further examined. For this purpose, LF adsorption isotherms were fitted to the log plot (log *B* versus log *F*) of the experimental adsorption isotherms obtained, according to the rebinding experiments described above. This was accomplished using the solver function in Microsoft Excel by varying the fitting parameters to reach a value of 1 for the coefficient of determination (*R*^2^) as described by Umpleby *et al*. [32].

### 3.7. Determination of the specificity of DS-MIP

The DS-MIP prepared was evaluated for its selectivity profile using the *D* and *S* templates, and various analogs such as salbutamol, isoproterenol, epinephrine, methyldopa and histamine as the substrates in saturation binding experiments. Weighed amounts of polymers (50 mg) were added to methanol/water (5 mL, 4:1, v/v) mixture containing the analyte of interest (5 μg/mL, 21.0–45.0 μM), or the pure solvent (blank, 5 mL), and stirred overnight at room temperature for equilibrium to be established. The polymer particles were then filtered, and the filtrate was analysed for free analyte content by fluorescence or UV spectroscopy [31], depending on the compound. The quantity of free analyte of interest in the solution was determined by reference to a calibration curve. The amount of bound drug was obtained by subtracting the amount of free drug from the total amount of the drug added. The ratio of the amount of the substrate bound by the MIP to that bound by the NIP was calculated. All experiments were performed in triplicate.

### 3.8. Determination of the binding-reactivity of the ergot derivatives

The binding reactivities of ergocryptine, ergocristine, ergocornine, ergonovine, agroclavine, pergolide and terguride, in an incubation medium were evaluated using a chromatographic assay. Briefly, DS-MIP particulates (2.5 mg/mL) were incubated between 0 and 13 h in 10-mL vials at room temperature by agitation with each ergot compound (2.5 μg/mL, 4.1–10.5 μM) in methanol/water (5 mL, 4:1, v/v). At pre-determined time intervals, the filtrate was analysed for the amount of unbound substance by HPLC (see below). The percentage of ergot bound to the polymer was plotted as a function of the incubation time. Each experiment was repeated three times.

A reversed phase HPLC-ECD detection method was used for assay of the ergots. A mobile phase comprising of a buffer (pH 7.4) and methanol (34:66, % v/v) was used for elution. The buffer composition was as follows: 15 mM KH_2_PO_4_, 3.75 mM sodium heptanesulfonate and 7.5 mM KCl aqueous solution adjusted to pH 7.4 by phosphoric acid. The analytical column was a Luna 5μ C_18_, 25 cm × 0.46 cm (Phenomenex, CA, USA). A flow-rate of 1.0 mL/min was used. The electrochemical detector was set at 0.8 V potential. Correlation coefficients for calibration curves for the ergots in the range 2–25 μg/mL (3.3–105 μM) were greater than 0.999. The sensitivity of detection was about 1.0 μg/mL (1.64–4.20 μM) and the reproducibility of the peak areas of analytes was more than 95%.

### 3.9. Competitive ligand binding assays for the tested ergot derivatives

Seven ergoline compounds (ergocryptine, ergocristine, ergocornine, ergonovine, agroclavine, pergolide and terguride) were assessed for their ability to displace *D* or *S* bound to DS-MIP. DS-MIP or NIP particles (25 mg) were mixed with 6.25 μg of either *D* (or *S*) and varying amounts of ergot (final concentration: 0.02 - 500 μg/mL or 0.03 - 890.20 μM) in the suspension solvent (4:1, v/v methanol/water) up to a final volume of 5 ml. The mixture was incubated for 8 h at room temperature, maintaining constant stirring. The changes in fluorescence intensity of *D* probe in solution were monitored at 320 nm following excitation at 279 nm; for the *S* probe in solution, monitoring was at 335 nm after excitation at 300 nm. The experimental signals were normalized as: *B/B*_î_, where *B* is the fluorescence intensity measured in the presence of increasing ergot concentrations and *B*_î_ is the signal in the absence of the ergot compound. The ratio of the normalized signal for MIP to that obtained for NIP was plotted as a function of the logarithm of the competitor concentration. Statistical analysis of concentration-response curves was performed by using the computer program GraphPad software [38], which calculates the lower and upper plateaus, the slope, and the *IC_50_*, concentration of an inhibitor producing 50% inhibition. An apparent inhibition constant (*K_i_*) value was calculated using eq (3):

(3) $$K_{i}=IC_{50}/(1+[\text{L}]/K_{d})$$

where *K_i_* = apparent inhibitor constant, [L] = free concentration of dopamine or serotonin, and *K_d_* = apparent dissociation constant of a given MIP for *D* (or *S*) [39]. All experiments were performed in triplicate.

### 3.10. Determination of the binding affinities of the ergot derivatives to the natural receptor

The equilibrium binding constants of the ergots on dopamine and serotonin receptors derived from rat hypothalamus was examined in a displacement assay using the added *D* and *S* as the molecular probes. The male-Wistar rat hypothalami receptors were isolated using a previously described procedure [40]. The receptor pellets were washed with 50 mM Tris HCl until the endogenous *D* and *S* are no longer found in the rinse as examined by the fluorescence method described in the binding study. The pellets were then re-suspended in 50 mM Tris HCl with 0.5 mM Na_2_EDTA, 0.1% Na ascorbate before use. The protein content of the receptor pellets was verified by using the procedure of Bradford [41]. Saturation experiments for the rat hypothalamus receptor were carried out by varying the concentration of *D* or *S* ligand in a 50 mM Tris HCl with 0.5 mM Na_2_EDTA and 0.1% Na ascorbate solution at 37 °C. The *D* and *S* binding at various concentrations was determined, using the fluorescence spectroscopic method for assay of the unbound probe as in the binding study. Scatchard analysis of the data showed good fits (linearity) to the binding isotherm for a single class of binding sites. The dissociation constant *(K_d_*) and receptor binding (*B*_max_) values were determined to be 0.53 ± 0.15 nM (mean ± SD, n = 3) and 1.50 ± 0.09 pmol/mg protein (mean ± SD, n = 3), respectively for the *D* ligand, and 0.11 ± 0.01 nM (mean ± SD, n = 3) and 1.11 ± 0.01 pmol/mg protein (mean ± SD, n = 3), respectively for the *S* ligand.

The ability of 6–8 concentrations of test ergot (0.1–500 μg/mL or 0.16–890.20 μM) for typical profiles) to displace 0.25 μmole *D* and *S* probes was measured in drug displacement studies. *D*/*S* binding was saturated to high affinity. To examine the equilibrium binding constant for the ergots, the unbound *D* and *S* probes was measured by fluorescence spectroscopy with the use of cation exchange resin to separate the protein bound and unbound probes. All experiments were performed four times using 50 mM Tris HCl with 0.5 mM Na_2_EDTA, 0.1% Na ascorbate as the medium, to which 10 mg of protein was added giving a final volume of 1 mL. The tubes were allowed to equilibrate for 30 min at 37 °C before filtering with a 0.45 μm cellulose acetate syringe filter (Whatman, NJ, USA) and were washed with two 5 mL ice-cold Tris-buffer. The filters were lyophilized and analysed for free probes. The supernatant aliquots were then passed through small columns of Amberlite CG-50 as previously reported [42]. The 3 mL of formic acid-ethanol (1:3, v/v) eluates containing the analytes were evaporated to dryness in a polyethylene vial and the residues reconstituted with methanol (5 mL) before assay using fluorescence spectrometry. The binding of *D* and *S* probes in the presence of the ergots was calculated and plotted to the ergot concentrations for determination of *IC_50_*. An apparent *K_i_* value was calculated in the same manner as described for the competitive ligand binding assay with DS-MIP.

### 3.11. Statistical analysis

Data are expressed as the mean ± Standard deviation (S.D.). The student’s *t*-test was applied, where necessary, to evaluate significance of difference. A value of p<0.05 was considered to be significant.

## 4. Conclusions

In summary, a molecularly imprinted polymer with dual dopamine/serotonin-like binding sites (DS-MIP) has been synthesised and evaluated for its recognition properties and its behavior in competitive assay for a series of ergot family alkaloids. Dopamine and serotonin selectivity as well as selectivity profile of the DS-MIP were demonstrated in saturation binding studies upon comparison with the non-imprinted polymer. This study demonstrates the ability of the various ergot alkaloids to displace bound *D* and *S* from the binding sites in competitive assays. In addition, the utilization of the DS-MIP in a competitive binding assay for ergot produced results which were comparable to those from a competitive immunoassay, obtained using dopamine/serotonin receptors derived from rat hypothalamus. The binding features described here will be utilized in the future with the incorporation of the DS-MIP into membranes for its study as a cell surface dopamine and serotonin receptor mimic, as well as its potential use in drug delivery.

## Figures and Tables

**Table 1.** Pore structure of the imprinted polymers and non-imprinted polymers (n=3).

*Polymer*	*Particle sizea)(μm)*	*Pore diameter (nm)*	*Pore volumeb)(mL/g)*	*BET Surface areab)(m^2^/g)*
NIP	29.10	52.21	0.25 (0.009)	201.9 (24.6)
DS-MIP	22.23	18.36	0.21 (0.006)	133.3 (17.5)
D-MIP c)	19.93	30.17	0.24 (0.014)	130.4 (34.4)
S-MIP	19.43	21.09	0.31 (0.005)	158.4 (14.8)

a). Mean values of three measurements are reported. The range of data was within ± 10% of the mean.

b). The micropore surface and pore volume (values in parenthesis) from a *t*-plot using Harkins-Jula average thickness.

c). The samples were from the same batches as those prepared in the recent work [31].

**Table 2.** Binding constants for the imprinted and non-imprinted polymers obtained by Scatchard analysis.

*Polymer*	*Ligand*	*B_max_* (μmol/g)	*K_d_*_1_ (mM)	*K_d_*_2_ (mM)
NIP	*D*	0.52 ± 0.17	1.21 ± 0.23	0.55 ± 0.23
	*S*	1.45 ± 0.60	3.33 ± 0.73	0.24 ± 0.09
DS-MIP	*D*	3.10 ± 0.52	0.073 ± 0.002	10.41 ± 0.21
	*S*	5.71 ± 0.32	0.027 ± 0.0003	1.67 ± 0.13
D-MIP	*D*	3.48 ± 0.70	0.094 ± 0.028	1.25 ± 0.60
	*S*	4.63 ± 1.32	0.196 ± 0.054	3.53 ± 1.02
S-MIP	*D*	3.34 ± 0.74	0.278 ± 0.092	10.01 ± 0.36
	*S*	4.65 ± 0.32	0.074 ± 0.012	14.49 ± 3.25

**Table 3.** Fitting parameters for the LF fits to the adsorption isotherms of MIPs.

	*DS-MIP*	*D-MIP*	*S-MIP*
*D*			
*N_t_*	3.64 ± 0.01 μmol/g	2.76 ± 0.19 μmol/g	10.27 ± 0.66 μmol/g
*a*	3.30 ± 0.01 mM^−1^	3.44 ± 0.05 mM^−1^	1.02 ± 0.12 mM^−1^
*K*_o_	4.17 ± 0.01 mM^−1^	4.48 ± 0.04 mM^−1^	1.03 ± 0.16 mM^−1^
*m*	0.84 ± 0.01	0.83 ± 0.03	0.75 ± 0.02
Limits of affinity distribution*	0.09–9.5 mM^−1^	0.09–9.5 mM^−1^	0.19–9.5 mM^−1^
*R^2^*	0.998	0.966	0.975

*S*			
*N_t_*	5.86 ± 0.01 μmol/g	46.54 ± 16.81 μmol/g	5.00 ± 0.37 μmol/g
*a*	4.73 ± 0.08 mM^−1^	0.07 ± 0.01 mM^−1^	4.02 ± 0.16 mM^−1^
*K*_o_	12.85 ± 1.09 mM^−1^	0.03 ± 0.001 mM^−1^	8.08 ± 0.36 mM^−1^
*m*	0.61 ± 0.02	0.82 ± 0.02	0.67 ± 0.01
Limits of affinity distribution*	0.21–25.0 mM^−1^	0.19–9.5 mM^−1^	0.20–14.18 mM^−1^
* R^2^*	0.976	0.993	0.997

*. These limits were calculated from the maximum and minimum values of free guest concentration (*F_max_* and *F_min_*) by the relationships *K_min_* =1/*F_ma_*_x_ and *K_max_* =1/*F_min_*.

**Table 4.** Displacement of *D* and *S* ligands from DS-MIP and rat hypothalamus by ergot derivatives.

*Ergot*	*K_i_* (μM)			
	*D ligand*		*S ligand*	
	*DS-MIPa)*	*Rat hypothalamusb)*	*DS-MIPa)*	*Rat hypothalamusb)*
Terguride	0.14 ± 0.01	0.00003 ± 0.000005	7.79 ± 0.17	0.0013 ± 0.0001
Pergolide	0.20 ± 0.01	0.0007 ± 0.0001	12.78 ± 0.09	0.0060 ± 0.0002
Agroclavine	0.40 ± 0.01	0.0015 ± 0.0002	5.31 ± 0.22	0.0012 ± 0.0001
Ergonovine	69.57 ± 0.51	0.0025 ± 0.0001	14.74 ± 0.29	0.0012 ± 0.0001
Ergocryptine	124.88 ± 1.22	0.0028 ± 0.0002	24.85 ± 0.69	0.0100 ± 0.0004
Ergocristine	141.08 ± 1.47	0.0076 ± 0.0001	19.33 ± 0.60	0.0068 ± 0.0002
Ergocornine	216.60 ± 2.59	0.0104 ± 0.0002	27.25 ± 0.78	0.0200 ± 0.0001

a). Displacement studies using 25 mg of polymer particulate with ergot concentration ranging from 0.02 to 500 μg/mL (0.03–890.20 μM) and 6.25 μg of *D* (or *S*) in a final volume of 5 mL (*D* = 6.60 μM, *S* = 5.90 μM) were performed at room temperature (28 ± 1 °C) as described in the Experimental Section. Mean ± SD, n = 3.

b). Displacement studies using 10 mg of protein with ergot concentration ranging from 0.1 to 500 μg/mL (0.16–890.20 μM) and 0.25 μmole of D (or S) in a final volume of 1 mL (250 μM) were performed at 37 °C as described in the Experimental Section. Mean ± SD, n = 4.
